# Microtransplantation improves the outcome of older patients with newly diagnosed acute myeloid leukemia: a single-center study with long-term follow-up

**DOI:** 10.3389/fonc.2026.1736302

**Published:** 2026-02-27

**Authors:** Juan Liu, Xiao-mei Huang, Xiao-shuang Li, Yan-yan Zhu, Pan-pan Lv, Ya-kun Yang, Tian Tian, Wan-jun Sun

**Affiliations:** 1 Department of Hematology, People’s Liberation Army (PLA) Rocket Force Characteristic Medical Center, Beijing, China; 2 College of Life Science and Bioengineering, Beijing Jiaotong University, Beijing, China

**Keywords:** a single-center study, acute myeloid leukemia, long-term follow-up, microtransplantation, older patients

## Abstract

**Background:**

Microtransplantation (MST) combines chemotherapy with infusion of HLA-mismatched granulocyte colony-stimulating factor-mobilized peripheral blood stem cells (G-PBSCs) without graft-versus-host disease (GVHD) prophylaxis, offering a potential therapeutic alternative for older acute myeloid leukemia (AML) patients.

**Methods:**

In this single-center study, 29 patients aged ≥60 years with newly diagnosed non-acute promyelocytic leukemia (AML) received MST between April 2008 and June 2021. Patients were stratified into two age cohorts: 60–70 years (*n* = 20) and >70 years (*n* = 9). Each MST course comprised induction or consolidation chemotherapy followed by G-PBSC infusion. Donor chimerism was monitored by the InDels assay. Endpoints included complete remission (CR), overall survival (OS), leukemia-free survival (LFS), relapse, non-relapse mortality (NRM), and safety. Competing risk analysis (Fine–Gray model) was used to evaluate the cumulative incidence of relapse and NRM. Transcriptomic profiling was performed in a subset of long-term survivors.

**Results:**

The median follow-up was 148.5 months (range 52.96–219.12). The CR rate was 86.2% (25/29), with no significant difference between age groups (90.0% vs. 66.7%, *p* = 0.290). The median OS was 20.00 months (range 1.00–205.00). Patients aged 60–70 years had significantly better OS than those >70 years (50.0% vs. 10.0%, *p* = 0.002). Similarly, LFS was higher in the younger group (45.0% vs. 10.0%, *p* = 0.015). Receiving >3 MST courses was associated with longer OS and LFS (both *p* < 0.001). Competing risk analysis showed a significantly higher cumulative incidence of relapse in the >70-year group (66.7% vs. 45.0%, *p* = 0.048). NRM did not differ significantly between groups (*p* = 0.13). GVHD occurred in one patient (3.4%). Transcriptomic analysis of four survivors revealed distinct gene expression profiles enriched in immune and hematopoietic pathways.

**Conclusion:**

MST is an effective and tolerable treatment for older AML patients, particularly those aged 60–70 years and those receiving more than three treatment courses. These results support MST as a viable alternative for older patients ineligible for intensive transplantation.

## Highlights

- MST is an alternative treatment for older AML patients.
- More than three courses of MST provide much better clinical outcomes for older AML patients.

## Introduction

Acute myeloid leukemia (AML) is most frequently diagnosed among people aged 65−74 years (1). Most older AML patients have very poor outcomes with a 5-year relative survival rate of only 12.5% (2, 3). Older AML patients usually can only choose non-intensive treatment without full-dose chemotherapy or transplantation (4, 5), and the median survival of older AML patients receiving low-dose chemotherapy is no longer than 16 weeks (6–9). Therefore, non-intensive dose treatments do not improve the outcome of older AML patients.

Recently, many new molecule-targeted drugs and transplantation innovations have greatly prolonged patients’ overall survival (10–13). Microtransplantation (MST) combines the infusion of HLA-mismatched donor granulocyte colony-stimulating factor-mobilized peripheral blood stem cells (G-PBSCs) with appropriately dose chemotherapy, but without a conditioning regimen or immunosuppressive drugs. Compared to the control group, MST statistically improves the complete remission (CR) rate to 80.0% and the 2-year leukemia-free survival (LFS) rate to 38.9% with rapid hematopoietic recovery in older AML patients (14). Some centers reported that MST could improve outcomes in AML patients (15–20), whereas other institutions did not find similar efficacy in treating AML patients (21, 22). Briefly, more studies need to be conducted to validate the efficacy and safety of MST in treating older AML patients.

To validate the efficacy and safety of MST, we evaluate the rates of CR, overall survival (OS), and LFS as well as graft-versus-host disease (GVHD) and treatment-related toxic effects among older AML patients receiving MST therapy at our single center.

## Materials and methods

### Patients and donors

A total of 29 patients (≥60 years) with newly diagnosed AML were enrolled between April 2008 and June 2021, with follow-up through 31 October 2025. Diagnosis was based on the World Health Organization criteria for primary AML or AML with a history of myelodysplastic syndrome, excluding acute promyelocytic leukemia (23, 24). Donors were healthy adults aged 18–55 years, with no history of malignancy or major chronic illness, and could be either related or unrelated to the patient. High-resolution HLA typing for HLA-A, HLA-B, HLA-C, HLA-DR, HLA-DQ, and HLA-DP alleles was performed using sequence-specific primer polymerase chain reaction (PCR-SSP; Invitrogen, Carlsbad, CA, USA). An HLA mismatch of 5/12 to 7/12 loci was considered acceptable (14, 17, 20).

The study was approved by the Institutional Review Board of PLA Rocket Force Characteristic Medical Center (Beijing, China). All patients and donors provided written informed consent in accordance with the Declaration of Helsinki.

### Microtransplantation protocol

Each MST course consisted of chemotherapy followed by infusion of G-CSF-mobilized peripheral blood stem cells (G-PBSCs). Induction chemotherapy included idarubicin (8–10 mg/m^2^) or mitoxantrone (6–8 mg/m^2^) for 3 days combined with cytarabine (100–150 mg/m^2^) for 7 days. G-PBSCs were infused 24 h after the last dose of cytarabine. Patients not achieving CR received a second induction cycle of the same regimen.

Post-remission therapy consisted of ≥2 consolidation courses, each comprising intermediate-dose cytarabine (1.0–1.5 g/m^2^ twice daily for 3 days) followed by G-PBSC infusion. Courses were typically spaced 3 months apart.

Patients aged 60–70 years without a high comorbidity index were preferentially considered for allogeneic hematopoietic stem cell transplantation (allo-HSCT). Those without a suitable donor or who declined allo-HSCT were offered MST.

No GVHD prophylaxis was administered. Prophylactic subcutaneous G-CSF and broad-spectrum intravenous antibiotics were given during neutropenia (absolute neutrophil count < 0.5 × 10^9^/L). Supportive care included transfusions and nutritional support as needed. Disease relapse during treatment was considered therapy failure, prompting either re-induction chemotherapy or discontinuation of MST.

**Figure 1:**
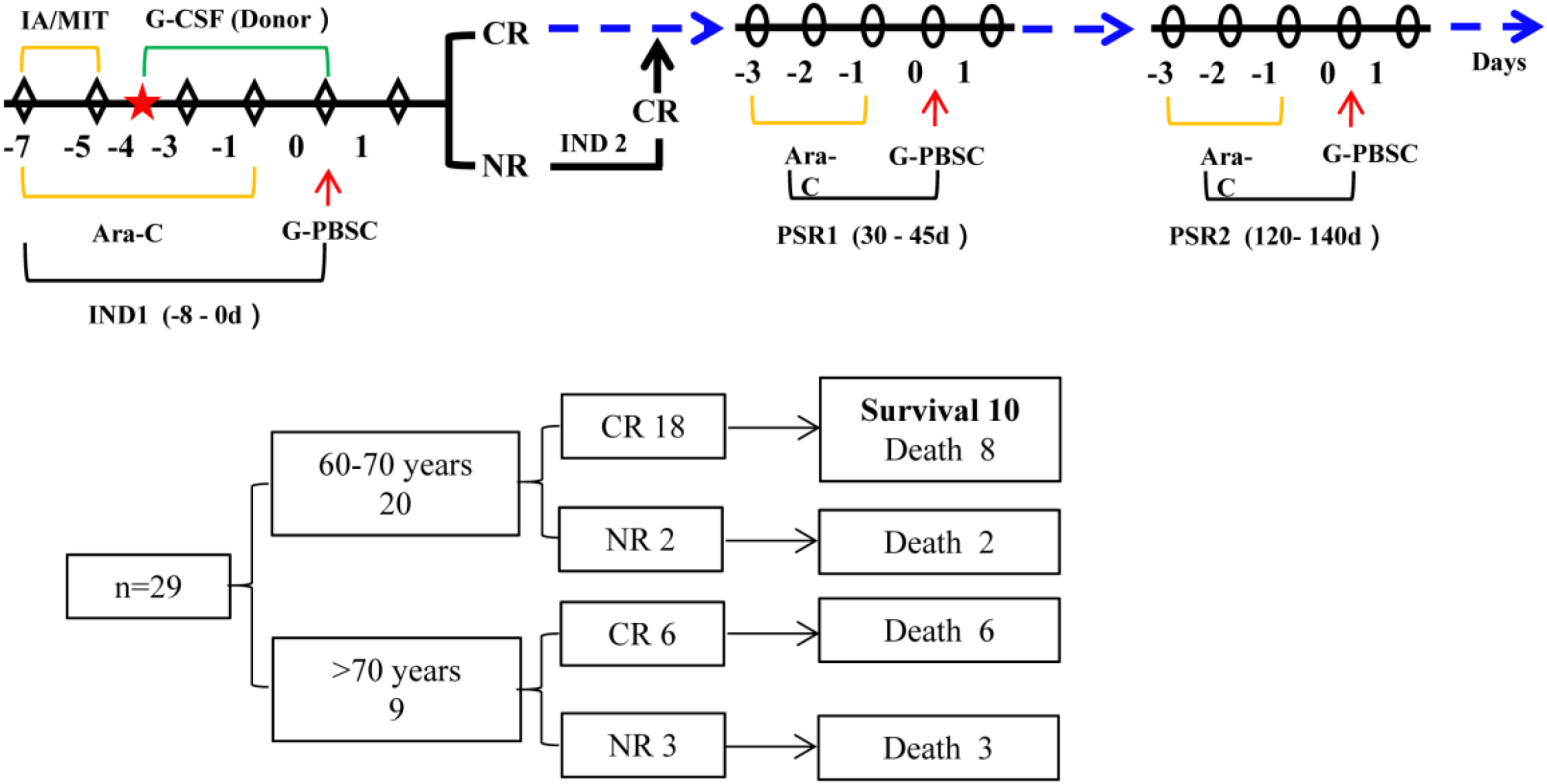
Protocol and results of MST for AML in older patients. Ara-C indicates cytarabine; MIT, mitoxantrone; IA, idarubicin hydrochloride; CR, complete remission; IND1, first induction chemotherapy; IND2, second induction chemotherapy; PSR1, first post-remission treatment; PSR2, second post-remission treatment.

The protocol and core clinical outcomes of MST for older AML patients are shown in **Figure 1**.

### Donor cell mobilization and collection

Donors received subcutaneous G-CSF (5 µg/kg twice daily for 5 days; Kirin Corp., Tokyo, Japan). When the leukocyte count reached approximately 50 × 10^9^/L, peripheral blood mononuclear cells were collected using a CS-3000S cell separator (Baxter, Deerfield, IL, USA). Collected G-PBSCs were aliquoted equally for each planned MST course; fresh cells were used for the first course, and cryopreserved (liquid nitrogen) cells were used for subsequent courses.

### Detection of donor microchimerism

The level of hematopoietic donor microchimerism was detected by the InDels assay, which had the ability to detect donor-specific cells at the 0.01% to 0.001% levels. The microchimerism detections were generally performed at the time of white blood cell count recovery before each new cycle of therapy. If the donor microchimerism was positive, we would perform the InDels assay to continually monitor donor microchimerism until negative after completing therapy.

### Study endpoints

According to the 2021 version of NCCN Guidelines Insights: Acute Myeloid Leukemia, primary endpoints such as CR, relapse, early death, cumulative incidence of relapse, non-relapse mortality (NRM), OS, and LFS were elaborated in detail as follows (25). GVHD was defined according to published criteria (26). CR required <5% bone marrow blasts, the absence of circulating blasts or extramedullary disease, neutrophil count >1.0 × 10^9^/L, platelet count >100 × 10^9^/L, and negative minimal residual disease by multiparameter flow cytometry. Relapse was defined as >5% marrow blasts, reappearance of blasts in the blood or extramedullary sites, or positive molecular markers. Early death referred to death within 4 weeks of chemotherapy initiation. Cumulative incidence of relapse was calculated from the CR date to relapse, with death without relapse as a competing risk. NRM included any death not due to relapse. OS was measured from diagnosis to death, and LFS was measured from CR to relapse or death from any cause. Censoring was applied at the last follow-up for patients alive, relapse-free, or lost to follow-up. Hematopoietic recovery time was defined as previously described (14): the starting point was the first of three consecutive days with ANC <2 × 10^9^/L and platelets <100 × 10^9^/L after chemotherapy; the endpoint was the first of three consecutive days with ANC >0.5 × 10^9^/L and platelets >30 × 10^9^/L.

### RNA sequencing and bioinformatics analysis

Total RNA was extracted from peripheral blood samples using TRIzol reagent (Invitrogen). RNA integrity and concentration were assessed using an Agilent 5400 Bioanalyzer. Poly(A)−tailed mRNA was enriched with Oligo(dT) magnetic beads, fragmented, and used for cDNA synthesis with M−MuLV reverse transcriptase. After second−strand synthesis, the cDNA was end−repaired, A−tailed, and ligated to adaptors. Libraries were size−selected (370–420 bp), amplified, and quantified with Qubit 2.0 and qPCR. Pooled libraries were sequenced on an Illumina NovaSeq 6000 platform to generate 150−bp paired−end reads. Base−calling and demultiplexing were performed using CASAVA, and raw reads were stored in FASTQ format.

### Differential expression and pathway analysis

Differentially expressed genes (DEGs) were screened using the DESeq2 R package (1.20.1). The resulting *p*-values were adjusted using Benjamini and Hochberg’s approach for controlling the false discovery rate. The log2 fold change (|log2 FC|) ≥1.0 and an adjusted *p*-value ≤0.05 was set as the threshold for significantly differential expression. KEGG is a database resource for understanding high-level functions and utilities of the biological system from molecular-level information, especially large-scale molecular datasets generated by genome sequencing and other high-throughput experimental technologies. We used the clusterProfiler R package (3.8.1) to test the statistical enrichment of differential expression genes in KEGG pathways.

### Statistical analysis

Categorical variables were compared using the chi-square test or Fisher’s exact test. Survival curves were generated with the Kaplan–Meier method and compared using the log-rank test. Univariate and multivariate Cox proportional hazards models were used to evaluate factors associated with survival endpoints. To account for competing risks between relapse and NRM, cumulative incidence functions were estimated using the Fine–Gray proportional subdistribution hazards model, and between-group comparisons were performed with Gray’s test. A two-sided *p*-value <0.05 was considered statistically significant. All analyses were conducted using SPSS version 21.0 (IBM, Armonk, NY, USA) and R software (version 4.0.3; R Foundation for Statistical Computing, Vienna, Austria).

## Results

### Patient and donor characteristics

A total of 29 patients (≥60 years) with newly diagnosed AML were included in the final analysis. The cohort was stratified into two age groups: 60–70 years (*n* = 20) and >70 years (*n* = 9). Donor age ranged from 18 to 55 years (mean 38.24 ± 6.16 years), and 82.76% (24/29) of donors were related to the recipients. No significant differences were observed between the two age groups in terms of patient sex, donor sex, donor–recipient sex matching, or HLA-mismatch status, or year of diagnosis (median 2013 in both groups, *p* = 0.489) (all *p* > 0.05). However, patients aged 60–70 years received significantly more than three courses of MST compared with those >70 years (90.00% [18/20] vs. 22.22% [2/9], *p* < 0.001) (**Table 1**).

**Table 1:** Patient and donor characteristics.

Variables	All patients (*n* = 29)	60–70 years (*n* = 20)	>70 years (*n* = 9)	*p*-value
Patient gender				0.412
Male, *n* (%)	11 (37.93)	9 (45.00)	2 (22.22)	
Female, *n* (%)	18 (62.07)	11 (55.00)	7 (77.78)	
Donor gender				0.700
Male, *n* (%)	15 (51.72)	11 (55.00)	4 (44.44)	
Female, *n* (%)	14 (48.28)	9 (45.00)	5 (55.56)	
Donor age (years), mean ± SD	38.24 ± 6.16	35.75 ± 4.52	43.78 ± 5.85	<0.001
Year of diagnosis, median (range)	2013 (2007–2020)	2013 (2007–2020)	2013 (2008–2016)	0.489
Donor/patient gender				0.107
Female to female, *n* (%)	8 (27.59)	3 (15.0)	5 (55.56)	
Female to male, *n* (%)	4 (13.79)	4 (20.0)	0 (0.00)	
Male to female, *n* (%)	10 (34.48)	8 (40.0)	2 (22.22)	
Male to male, *n* (%)	7 (24.14)	5 (25.0)	2 (22.22)	
Relation to donor				0.153
Unrelated, *n* (%)	5 (17.24)	5 (25.00)	0 (0.00)	
Related, *n* (%)	24 (82.76)	15 (75.00)	9 (100.00)	
Donor/recipient with HLA-mismatched loci				0.382
5/10, *n* (%)	22 (75.86)	14 (70.0)	8 (88.89)	
6/10, *n* (%)	7 (24.14)	6 (30.0)	1 (11.11)	
Courses of MST				<0.001
>3, *n* (%)	20 (68.97)	18 (90.0)	2 (22.22)	
1–3, *n* (%)	9 (31.03)	2 (10.0)	7 (77.78)	

### Response to induction chemotherapy

The overall CR rate was 86.21% (25/29). Two patients achieved CR after two cycles of induction chemotherapy. The CR rates were 90.00% (18/20) in the 60–70-year group and 66.67% (6/9) in the >70-year group, with no statistically significant difference between the groups (*p* = 0.290) (**Table 2**).

**Table 2:** Patient outcomes.

Variables	All patients (*n* = 29)	60–70 years (*n* = 20)	>70 years (*n* = 9)	*p*-value
MNC × 10^8^/kg, M (Q_1_, Q_3_)	3.57 (2.66, 4.23)	3.48 (2.65, 4.23)	3.73 (2.92, 3.98)	0.887
CD34^+^ cells × 10^6^/kg, M (Q_1_, Q_3_)	1.97 (0.74–4.29)	2.40 (0.93–4.29)	1.54 (0.74–3.16)	0.120
CD3^+^ cells × 10^8^/kg, M (Q_1_, Q_3_)	0.98 (0.80, 1.60)	0.99 (0.80, 1.74)	0.98 (0.89, 1.44)	0.850
Induction regimen, MA/IA, *n* (%)	30 (100.00)	20 (100.00)	9 (100.00)	0.999
Overall CR, *n* (%)	25 (86.21)	18 (90.00)	6 (66.67)	0.290
Total death, *n* (%)	21 (72.41)	12 (60.00)	9 (100.00)	0.049
Early death, *n* (%)	4 (13.79)	1 (5.00)	3 (33.33)	0.076
Causes of death				0.972
Relapse, *n* (%)	11 (37.93)	6 (30.00)	5 (55.55)	
Severe infection, *n* (%)	4 (13.79)	2 (10.00)	2 (22.22)	
Organ failure, *n* (%)	14 (48.28)	7 (35.00)	7 (77.78)	
OS, *n* (%)				0.154
1-year OS, *n* (%)	19 (65.52)	16 (80.00)	3 (33.33)	
3-year OS, *n* (%)	13 (44.83)	13 (65.00)	0 (0.00)	
5-year OS, *n* (%)	9 (31.03)	9 (45.00)	0 (0.00)	
LFS, *n* (%)				0.563
1-year LFS, *n* (%)	17 (58.62)	16 (80.00)	1 (11.11)	
3-year LFS, *n* (%)	10 (34.48)	10 (50.00)	0 (0.00)	
5-year LFS, *n* (%)	9 (31.03)	9 (45.00)	0 (0.00)	

### Overall mortality, early mortality, and causes of death

The overall mortality rate was 72.41% (21/29). Early mortality (death within 4 weeks after chemotherapy initiation) occurred in 13.79% (4/29) of patients. Mortality was significantly lower in the 60–70-year group than in the >70-year group (60.00% [12/20] vs. 100.00% [9/9], *p* = 0.049). Causes of death included relapse (37.93% [11/29]), severe infection (13.79% [4/29]), and organ failure (48.28% [14/29]), with no significant difference between the age groups (**Table 2**).

### Hematopoietic recovery and adverse events

The median time to neutrophil recovery was 12 days, and to platelet recovery, it was 14 days after induction chemotherapy. Adverse events included cardiac failure (44.83% [13/29]), arrhythmia (20.69% [6/29]), elevated liver enzymes (79.31% [23/29]), and gastrointestinal symptoms (nausea 65.52% [19/29], vomiting 58.62% [17/29], and diarrhea 44.83% [13/29]). Sepsis was documented in 17.24% (5/29) of patients. The incidence of adverse events did not differ significantly between the two age groups (**Supplementary Table 1**).

### Overall survival and leukemia-free survival

**Figure 2:**
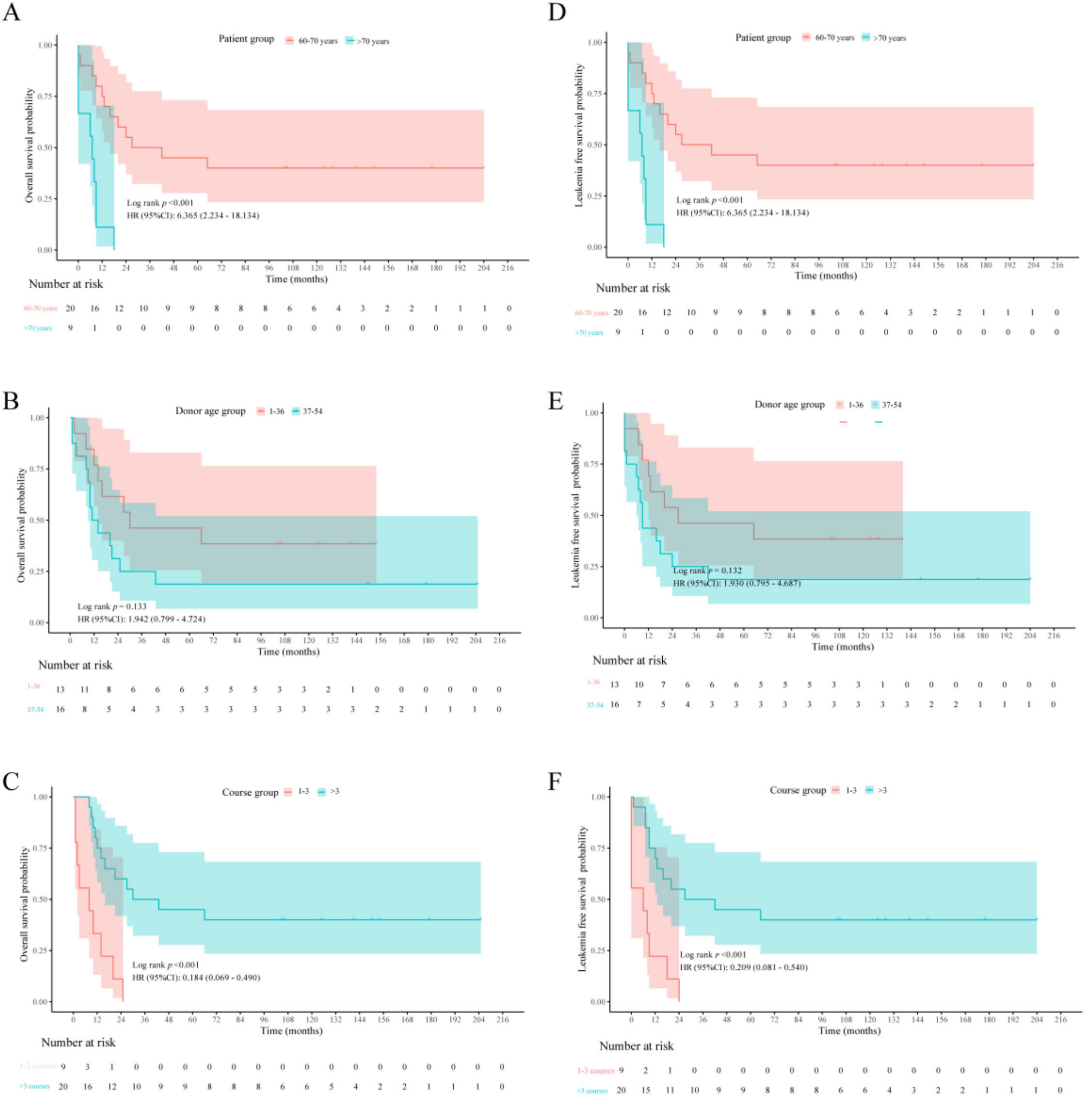
Survival outcomes of older AML patients after MST. **(A–C)** Overall survival and **(D–F)** leukemia-free survival analyses were performed by dividing patients into two groups according to patient age **(A, D)**, donor age **(B, E)**, or the number of courses of MST therapy **(C, F)**.

The median follow-up time was 148.47 months (range: 52.96–219.12 months), and the median OS was 20.00 months (range: 1.00–205.00). The 1-year overall survival rate was significantly higher in the 60–70-year group than in the >70-year group (80.00% [16/20] vs. 33.33% [3/9], *p* = 0.032) (**Table 2**). Patients who received more than three courses of MST had significantly longer OS and LFS (both *p* < 0.001) (**Figures 2A, C, D, F**). Donor age showed no significant association with OS or LFS (**Figures 2B, E**). It is important to note that this association should be interpreted with caution, as the ability to receive multiple courses inherently requires patients to survive longer and maintain remission in the first place. Thus, this finding may partly reflect a selection bias toward patients with more favorable disease biology and treatment tolerance, rather than solely a direct causal effect of the additional cycles.

### Relapse and non-relapse mortality

**Figure 3:**
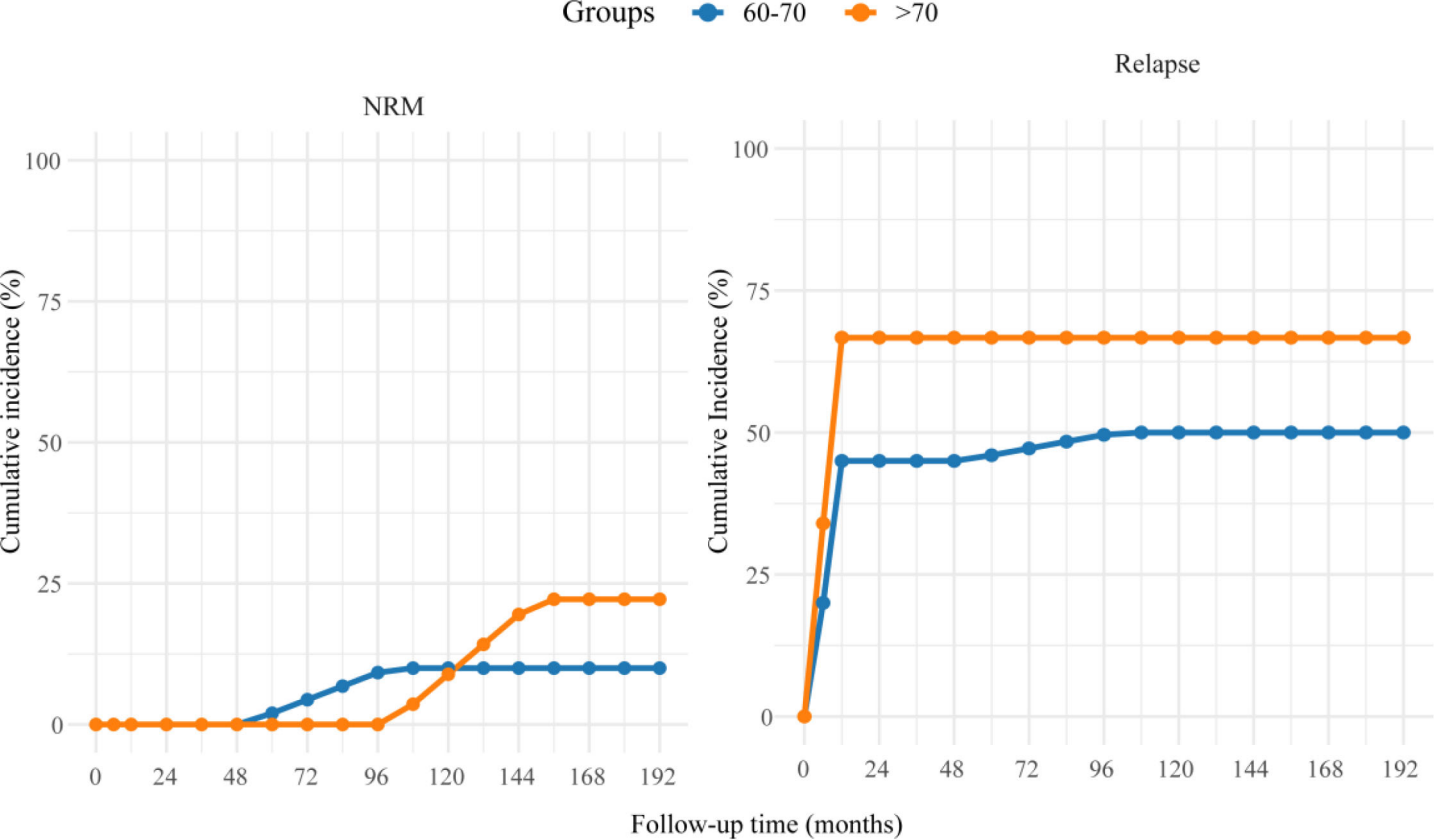
Cumulative incidence curves of relapse and non-relapse mortality (NRM) in older AML patients undergoing microtransplantation. Cumulative incidence of NRM (left) and relapse (right) was estimated using competing risk analysis (Fine–Gray model) and stratified by age group: 60–70 years (*n* = 20, blue lines) and >70 years (*n* = 9, orange lines). Relapse and NRM were treated as mutually competing events. The cumulative incidence of relapse was significantly higher in the >70-year group compared with the 60–70-year group (Gray’s test, *p* = 0.048), while the difference in NRM between groups did not reach statistical significance (*p* = 0.13).

Competing risk analysis (Fine–Gray model) revealed a significantly higher cumulative incidence of relapse in the >70-year group compared with the 60–70-year group (66.67% [6/9] vs. 45.00% [9/20] at 2 months, *p* = 0.048). NRM remained low during the first 4 years in both groups, then gradually increased, reaching 10.00% (2/20) in the 60–70-year group and 22.22% (2/9) in the >70-year group by 156 months; however, the difference between groups was not statistically significant (*p* = 0.13) (**Figure 3**; **Supplementary Table 3**).

### Donor microchimerism and graft-versus-host disease

A total of 96.6% patients had negative donor microchimerism. Only one 62-year-old female patient was diagnosed with severe acute GVHD with high fever, rash, diarrhea, and severe hyperbilirubinemia, and a mixed donor chimerism was detected after 4 courses of MST therapy. The donor of the patient was her 28-year-old daughter. The patient was infused with MNC 3.84 × 10^8^/kg, CD34^+^ 3.21 × 10^6^/kg, and CD3^+^ 0.76 × 10^8^/kg cells in each course of MST therapy. The chimerism rate increased from negative to 7.973%, and the rate shot up to 25.809% for 1 week. The patient failed to respond to anti-GVHD treatment and died of multiorgan failure at day 44 after the fourth MST.

### Univariate Cox regression analyses

Univariate Cox proportional regression analysis showed that patient age and the number of courses of MST therapy might be the factors of OS (**Supplementary Figure 1A**). Patient age, donor gender, donor age, and the number of courses of MST therapy might be the factors of LFS (**Supplementary Figure 1B**).

### Transcriptomic profiling of AML patients after MST

**Figure 4:**
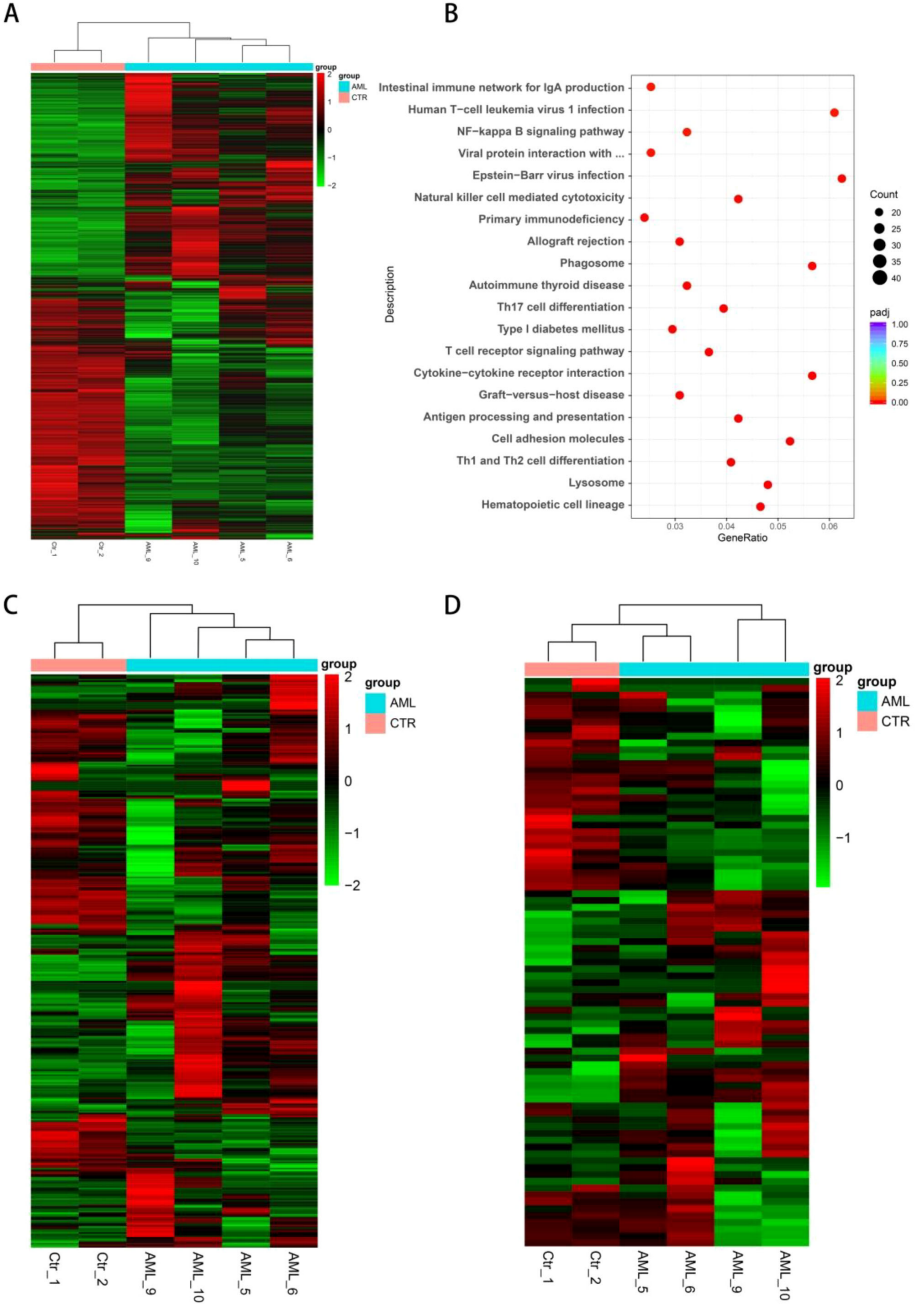
RNA sequencing analysis of peripheral blood in AML patients and healthy individuals. **(A)** Heatmaps depicting all differentially expressed genes in AML and control groups. **(B)** KEGG analysis of DEGs in different cell functions. **(C)** Heatmaps depicting differentially expressed genes in immune-related functions. **(D)** Heatmaps depicting differentially expressed genes of AML markers.

RNA sequencing analysis was utilized to evaluate the status of AML patients who received MST on the mRNA level. We randomly chose four still alive (surviving) AML patients after MST and two healthy individuals as the control group. The clinical information of these four patients is listed in **Supplementary Table 2**. The heatmap demonstrates the transcriptional changes in cellular gene expression in the peripheral blood of each patient and shows the DEGs. In total, 1,579 expressed genes were detected. Compared with two healthy individuals, 883 genes were found to be significantly upregulated in four AML patients, and 696 genes were downregulated (**Figure 4A**). KEGG analysis was performed on the DEGs and revealed expression changes in the genes associated with hematopoietic cell lineage, lysosome, Th1 and Th2 cell differentiation, cell adhesion molecules, and some immune-related pathways (**Figure 4B**). Furthermore, we filtered some immune-related genes and some AML marker genes from DEGs to compare in AML and healthy individuals (**Figures 4C, D**). These genes are listed in **Supplementary Tables 4** and **5**. As demonstrated in these heatmaps, patients 5 and 6 are similar (shorter LFS and OS), and patients 9 and 10 (longer LFS and OS) are similar in DEGs, while these two groups still have significant differences compared with the healthy group.

## Discussion

The current study summarized the experience of MST therapy for newly diagnosed AML patients aged 60 to 85 years. As we all know, complete remission and hematopoietic recovery as soon as possible are vital in treating AML (27–29). Our data showed that the overall CR rate was 83.3% (25 of 30), and OS rates at 1 and 3 years were 66.7% and 36.7%, respectively (**Table 2**), indicating an equivalent efficacy compared to the results of other MST reports (14, 17). Additionally, the median neutrophil recovery time and the platelet recovery time were 12 and 14 days in MST, respectively, which were also similar to other MST studies (14, 17). Therefore, our experience presented that MST therapy could be an alternative treatment for older AML patients.

Patents’ age was still an important prognostic factor in this study. Although there was no significant difference in overall CR rate between the two age groups (90% vs. 70.0%, *p* = 0.3), the younger group (aged 60–70 years) had a much lower death rate, longer OS time, and longer LFS time than the older group (aged > 70 years). In the >70-year group of the study, the median OS time was 10 months and the 1-year OS rate was 40.0%. Similarly, Kantarjian H et al. also reported that intensive chemotherapy did not benefit most older patients (age 70 years or older) with AML (median survival: 4.6 months, 1-year OS rate: 28%) (28). Although MST is a novel tool for treating AML patients aged >70 years, the chemotherapy component in MST may be adjusted with tolerable medicines, such as venetoclax and azacitidine.

As in other MST studies, our patients were suggested to receive at least two courses of MST for post-remission therapy. Guo Mei et al. reported that three courses of MST as post-remission therapy for AML significantly improved the 6-year LFS and OS rates in the low-risk and intermediate-risk groups (20). In another study, older AML patients who achieved CR receiving two to three courses of MST as post-remission therapy also achieved a high 2-year OS rate (17). Our study also found that more than three courses of MST therapies could prolong the OS time and LFS time of older AML patients. Therefore, to a certain extent, more courses of MST therapy may provide longer-term benefit for older AML patients.

The infusion of donor stem cells might induce antileukemic responses from MST therapy (30). In MST therapy, the incompletely destroyed immune system of the patient could reject most donor cells. Therefore, a kind of microchimerism exists in patients by receiving sequential MST therapy in post-remission treatment. Microchimerism was reported to be a potential reason for MST anti-leukemia (31, 32). Additionally, an MST mouse model has demonstrated that G-PBSC infusion may stimulate recipient-derived T-cell responses for indirect antitumor effect (33). However, one patient in our study developed serious acute GVHD and died of multiorgan failure. If patients have persistent high fever, rash, diarrhea, hepatic injury, and chimerism higher than 1% at 1 week after stem cell infusion, we should pay close attention to the risk of GVHD (17). At this time, we need to monitor the cytokines IL-6, IL-8, sTNFR1, sST2, Reg3α, and Elafin to predict GVHD risk. Generally, steroid therapy and CD25mab will be adopted, and the intermission of microtransplantation will be extended for patients when there is rising chimerism.

Azacitidine has been primarily used to treat AML patients older than 60 years or those who are unable to accept intensive conventional chemotherapy. Venetoclax combined with azacitidine has been usually used to treat older adults with primary or secondary AML who were ineligible for conventional chemotherapy (34). The rates of CR or CR with incomplete hematologic recovery (CRi) were 54% for the low-dose cytarabine/venetoclax regimen and 67% for the azacitidine/venetoclax doublet, with a significant extension in OS to a median of 10–18 months (35). If we combine azacitidine and/or venetoclax with stem cell infusion as a new microtransplantation regimen in the treatment of older AML patients, the drug resistance rate may be reduced, and patients may obtain higher CR rates and longer overall survival.

### Limitations and future directions

In the interpretation of our findings, several limitations must be considered. First, the observed association between the number of MST cycles and survival outcomes is susceptible to “immortal time bias,” as patients must survive and remain in remission to receive subsequent courses. Therefore, this association likely reflects, in part, the selection of patients with favorable disease biology and treatment tolerance rather than solely a direct causal effect of treatment intensity. Second, the single-center, retrospective design and the small sample size, especially in the >70-year subgroup, limit the generalizability of our results.

### Conclusion

Our study suggests that MST is an effective and tolerable strategy, particularly for AML patients aged 60–70 years. The association between receiving more than three MST courses and superior long-term survival highlights the potential importance of sustained treatment in eligible patients.

Future prospective, multicenter studies with larger cohorts are warranted to validate these findings. Moreover, investigating optimized, lower-intensity chemotherapy backbones (e.g., combining hypomethylating agents with venetoclax) within the MST platform and employing statistical methods like time-dependent analysis will be crucial to better delineate the specific contribution of treatment intensity to outcomes in older AML patients.

## Data Availability

The original contributions presented in the study are included in the article/**Supplementary Material**. Further inquiries can be directed to the corresponding authors.

## Supplementary material

Supplementary Figure 1 Univariant analyses of outcomes of older AML patients after MST. Univariant analyses of OS **(A)**, LFS **(B)**, cumulative incidence of relapse **(C)** and NRM **(D)** after MST therapy.

Supplementary Table 1 Adverse Events.

Supplementary Table 2 Clinical characteristics of 10 patients with long OS and 2 controls.

Supplementary Table 3 Cumulative incidence of relapse and non-relapse mortality (NRM) estimated by competing risk analysis (Fine-Gray model) in microtransplanted AML patients.
