# Supplementary figures and images for: Microtransplantation improves the outcome of older patients with newly diagnosed acute myeloid leukemia: a single-center study with long-term follow-up
Source: Front Oncol. 2026 Feb 27;16:1736302. doi: 10.3389/fonc.2026.1736302 (PMC12982073; doi:10.3389/fonc.2026.1736302)

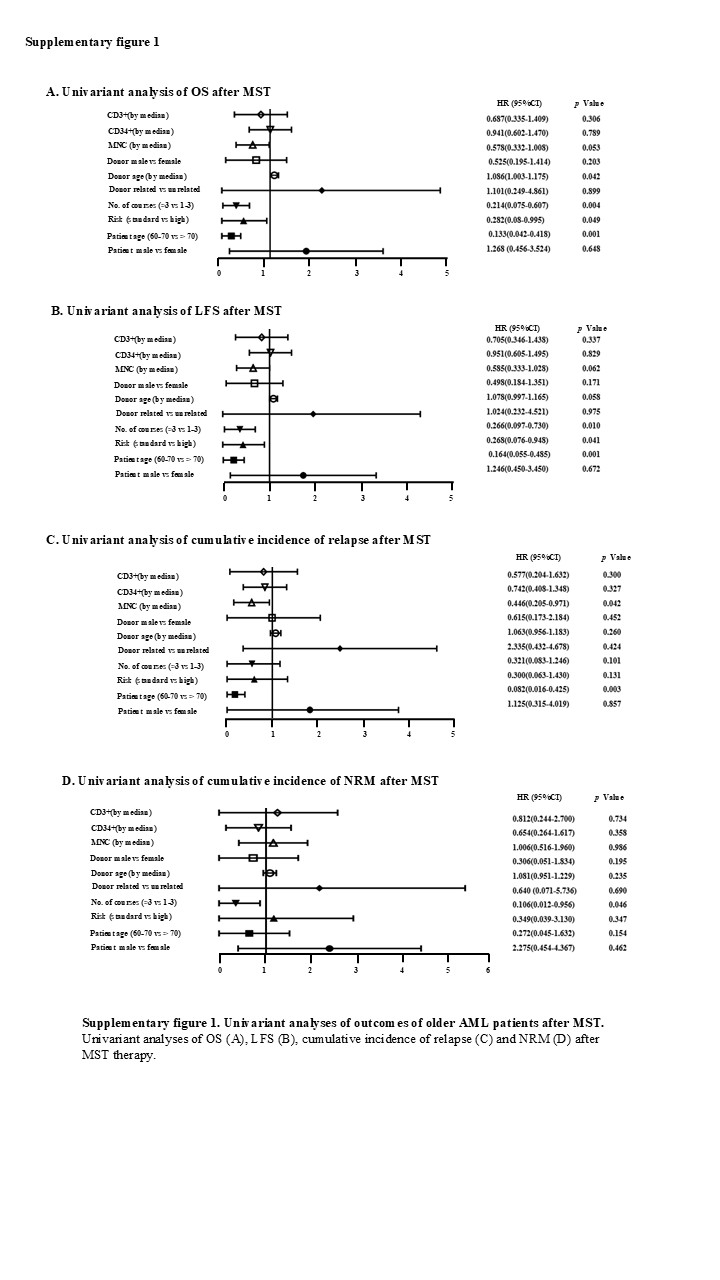

Supplement: Supplementary Figure 1 — Univariant analyses of outcomes of older AML patients after MST. Univariant analyses of OS (A), LFS (B), cumulative incidence of relapse (C) and NRM (D) after MST therapy. [file Image1.jpeg]
